# Negative associations of age and lifestyle factors with the antibody response to the COVID-19 vaccine BNT162b2 in health workers from Spain

**DOI:** 10.3389/fimmu.2025.1590939

**Published:** 2025-05-30

**Authors:** Luis Sarabia de Ardanaz, Esther Serrano-Conde, Ana Fuentes, Alba Leyva, Federico García, Pilar Requena

**Affiliations:** ^1^ Universidad de Granada, Departamento de Medicina Preventiva y Salud Pública, Granada, Spain; ^2^ Servicio de Reumatología, Hospital Universitario de Jaén, Jaén, Spain; ^3^ Servicio de Microbiología, Hospital Universitario Clínico San Cecilio, Granada, Spain; ^4^ Instituto de Investigación Biosanitaria de Granada (ibs.GRANADA), Granada, Spain; ^5^ Centro de Investigación Biomédica en Red de Enfermedades Infecciosas (CIBERINFEC), Madrid, Spain; ^6^ Vircell, S.L., Granada, Spain; ^7^ Centro de Investigación Biomédica en Red de Epidemiología y Salud Pública (CIBERESP), Madrid, Spain

**Keywords:** COVID-19, SARS-CoV-2, vaccines, BNT162b2, anti-spike IgG, neutralizing antibodies

## Abstract

**Introduction:**

Despite the high efficacy of the anti-coronavirus disease 2019 (COVID-19) BNT162b2 vaccine (Comirnaty^®^, Pfizer-BioNTech), variability in the antibody titers following vaccination has been described. However, little is known about the risk factors that are associated with a poorer antibody response to the BNT162b2 vaccine.

**Methodology:**

We studied the determinants of the humoral response to the anti-COVID-19 vaccine BNT162b2 in 200 healthcare workers followed up for 2 years. Serum samples were tested for the anti-spike immunoglobulin G (IgG) levels and neutralizing antibody titers against selected severe acute respiratory syndrome coronavirus 2 (SARS-CoV-2) variants at different time points after primary and booster vaccinations. Anthropometric data and clinical and lifestyle information were also collected. Statistical analyses consisted of linear and logistical regressions for point estimations and the Mann–Whitney, Friedman, and generalized estimating equations for repeated measures.

**Results:**

After the primary vaccination, the antibody titers and the percentage of seroconverted individuals peaked at 5 weeks but declined after 1 year; however, they remained high after the booster administration. After the first dose of the vaccine, negative associations of the anti-spike IgG levels with age (*β* = −0.01, 95%CI = −0.03 to −0.003), smoking habit (*β* = −1.08, 95%CI = −1.70 to −0.46), and alcohol consumption (*β* = −1.43, 95%CI = −2.20 to −0.65) were found. With regard to the booster vaccine, the following associations were retained in the stepwise multivariate model: anti-Delta neutralizing antibodies with hip circumference (OR = 1.07, 95%CI = 1.01–1.12, *p* = 0.008), anti-Delta-K antibodies with hip circumference (OR = 1.06, 95%CI = 1.01–1.11, *p* = 0.007), and anti-Omicron antibodies with the Mediterranean diet score (OR = 0.74, 95%CI = 0.58–0.96, *p* = 0.023).

**Conclusion:**

Lifestyle habits and age had an association with the humoral response to the BNT162b2 vaccine.

## Introduction

From the beginning of the coronavirus disease 2019 (COVID-19) pandemic, severe acute respiratory syndrome coronavirus 2 (SARS-CoV-2) infection has remained an extraordinary health risk based on the reported rates of infection, disease, and mortality (1). Throughout the pandemic, several variants of the virus have emerged, and some of them have been designated by the World Health Organization as “variants of concern,” i.e., Alpha, Beta, Gamma, Delta, and Omicron.

The genome of SARS-CoV-2 encodes several proteins. Among them, the spike (S) glycoprotein is expressed in the virus surface and is responsible for the viral attachment, the fusion, and the endosome-mediated host entry (2). Therefore, some of the antibodies generated against this protein have the ability to block the entry of the virus into the target cells and are so-called neutralizing antibodies.

Mass vaccination plays a crucial role in the prevention and management of infectious diseases. In the case of SARS-CoV-2, the S protein is the target of most commercialized vaccines. This is the case with BNT162b2 (Comirnaty^®^, Pfizer-BioNTech), which contains messenger RNA (mRNA) encoding the S protein, and in clinical trials provided approximately 95% protection against COVID-19 when two doses were administered (3). Research indicates that the maximum humoral immune response occurs between 21 and 28 days following the administration of the second vaccine dose. Subsequently, there is a gradual reduction in the serum antibody levels at 4–6 months post-vaccination, irrespective of factors such as age, gender, initial serostatus, or the presence of other health conditions (4). However, we and others have shown variability in the antibody titers after vaccination or natural disease, including individuals who did not develop a humoral response at all (5–7). This does not necessarily mean a lack of immunity since cellular responses could equally confer protection in the absence of antibodies. However, it is necessary to examine what the determinants of this lower humoral response may be. It has been noted that the levels of neutralizing antibodies or their decline is associated with the comorbidities of the patient. Thus, individuals of older age and those with immunosuppression produced a significantly lower antibody response after the first vaccine dose compared with healthy adults (8). This has also been observed in obese individuals regardless of sex, previous infection status, or the time elapsed since the last vaccination dose (9). However, in these studies, the neutralizing antibodies for variants other than the wild type were not considered. Furthermore, additional socio-demographic, clinical, or lifestyle factors could affect the immune response after vaccination.

Therefore, the aim of this study was to assess the determinants of a lower humoral response to the BNT162b2 vaccine, analyzed as the total anti-S immunoglobulin G (IgG) levels and neutralizing antibodies against different variants.

## Materials and methods

### Study design and population

This is an ambispective internal comparison cohort study performed in two stages. Initially, 147 workers (cohort 1) from the Hospital Universitario Clinico San Cecilio who received two doses of BNT162b2 and were under 65 years of age were recruited. The study participants were enrolled in January 2021, in order of arrival to the vaccination event, and were followed up until December 2021. In a second stage and coinciding with the administration of the third dose of vaccine in December 2021, participants were invited to continue the follow-up for an additional year and to reply to an auto-administered questionnaire, with 99 out of 147 individuals providing their consent. In addition, 54 more individuals were recruited to compensate for the losses at follow-up (estimated to be 10% every 6 months due to the high turnover of healthcare personnel in the hospital). Those 99 plus 54 individuals comprised cohort 2.

### Study samples and data collection

Blood samples were collected from the initial cohort at the following times: during administration of the first dose (*t*_0_) and the second dose (3 weeks later, *t*_1_), as well as 5 weeks (*t*_2_), 3 months (*t*_3_), and 8–9 months (*t*_4_) after administration of the first dose. Moreover, coinciding with the administration of the third dose of the vaccine, additional samples were collected (*t*_5_), as well as 1 month (*t*_6_), 4–6 months (*t*_7_), and 9 months (*t*_8_) after the third dose. For the 54 additional subjects recruited in the second stage, only samples collected from *t*_5_ to *t*_8_ were obtained.

Blood samples were obtained at each time point, and serum was obtained by centrifugation of the blood samples at 600 × *g* for 5 min, aliquoted, and kept at −20°C until use.

At *t*_5_, an interview was performed and anthropometric data [body mass index (BMI) and waist and hip perimeters] were determined by a dietician–nutritionist. Moreover, a questionnaire was auto-filled with regard to information about the times of administration of the first and third doses. The information collected included the following: 1) socio-demographic, including sex, age, and level of education; 2) lifestyle habits, with the 14-item PREDIMED adherence to Mediterranean diet (MedD) questionnaire (10), smoking habit (collected into categories of never smoker, ex-smoker, or active smoker), and alcohol consumption (informed as never, low consumption of ≤7 alcoholic drinks spread throughout the week or concentrated in 1–2 days, or high consumption of >7 drinks spread throughout the week or concentrated in 1–2 days); and 3) auto-reported clinical information with regard to prevalent diseases at the time of vaccination, classified as a) immune-mediated diseases (i.e., hematological neoplasms, primary or secondary immunodeficiencies, autoimmune diseases, and/or transplant recipients), b) cardiometabolic diseases (e.g., heart diseases, dyslipidemia, and/or diabetes mellitus, and c) other diseases (e.g., cancer and/or respiratory diseases).

Infections prior to vaccination were auto-reported by the study subjects.

### Antibody determination

The serum anti-S IgG antibodies were analyzed in all samples using the COVID-19 VirClia^®^ IgG mono test (Vircell, S.L., Granada, Spain) following the manufacturer’s instructions. This is a qualitative indirect chemiluminescent immunoassay with a manufacturer-declared sensitivity of 98%. Individuals were considered as seroconverted when their antibody levels were higher than the mean plus 3 standard deviations of the levels observed before vaccination.

A full-virus assay was used to test the neutralization capacity of the serum of the study participants against the wild type and the Alpha (B.1.1.7), Beta (B.1.351), Delta (B.1.617), Delta_K (B.1.617+E484K), and Omicron (B.1.1.529) variants of SARS-CoV-2. Briefly, 70 µl of serum was twofold serially diluted in a culture medium with 2% fetal bovine serum, starting at 1:20 dilution until 1:2,560, and added into microplates. Thereafter, 70 µl of the cell culture medium containing 100× of the median tissue culture infectious dose of the virus was added to every well containing the diluted sera of the participants. The plates were incubated at room temperature for 1 h, and 100 µl of each mixture of serum plus virus was added into a plate containing Vero E6 cells. After a 5-day incubation period at 37°C in 5% CO_2_, the full cytopathic effect was evaluated by microscopic examination. The highest serum dilution that completely inhibited the cytopathic effect was considered as the neutralization titer.

Samples below or over the limit of detection were assigned the value of the detection limit.

### Statistical analysis

A descriptive analysis of the variables was carried out. The variables were presented as follows: mean ± standard deviation (SD) for normally distributed continuous variables, median for non-normally distributed variables, and percentages and frequencies for categorical variables.

The differences in the levels of anti-S between time points (*t*_0_ − *t*_5_ and *t*_5_ − *t*_8_) were analyzed by means of the Kruskal–Wallis test followed by individual comparisons *versus t*_0_, corrected with the Dunn’s test for multiple comparisons. It was not possible to perform a paired test due to the losses at follow-up. The differences in the neutralizing antibody titers before/after the booster administration (*t*_5_ − *t*_6_) were assessed with the Wilcoxon matched-pairs signed-rank test, as in this case there were no losses at follow-up. Furthermore, the differences between the different variant neutralizing antibody titers were analyzed with the Friedman’s test followed by Dunn’s multiple comparisons test using the Omicron variant as a reference. These analyses were only performed here for the Omicron, Delta, and Delta_K variants after the booster administration as the analysis of the wild-type and the Alpha and Beta variants after primary vaccination had already been published in a previous article (5).

Univariate linear regression models for the anti-S IgG levels and univariate ordinal logistic regression models for the neutralizing antibody titers were estimated to identify the determinants of antibody levels after the primary and booster vaccinations. Beta coefficients (*β*) or odds ratios (ORs), as well as the 95% confidence intervals (CIs) and *p*-values, were estimated. Subsequently, those variables with *p* < 0.2 were included in the stepwise multivariate model.

Finally, generalized estimating equation models for repeated measures were utilized to evaluate the determinants of the average antibody levels over the follow-up. Initially, univariate analyses were conducted, and variables with *p* < 0.2 were subsequently included in the multivariable analysis.

Statistical significance was indicated as *p* < 0.05. Stata program v.17 and GraphPad Prism v.10.2.3 were used for the statistical analysis and graphs.

## Results

### Description of the study cohort

As shown in **Table 1**, the overall mean age was 46.52 years (SD = 11.86), 20.90% were men, and most individuals completed higher education studies. The average BMI was 25.37, indicating a slightly overweight population. With regard to lifestyle habits, the adherence to MedD was high, and most study subjects did not smoke or drink alcohol.

**Table 1:** Baseline demographic and clinical variables.

	Cohort 1	Cohort 2	Total
Age, mean (SD) [*n*]	45.31 (11.92) [147]	47.71 (11.69) [153]	46.52 (11.86) [200]
Sex (men), *n* (%)	33 (22.45)	30 (19.48)	42 (20.90)
Higher education studies (yes), *n* (%)	70 (79.55)	104 (77.04)	107 (76.98)
Waist circumference, mean (SD) [*n*]	83.87 (12.49) [88]	83.66 (12.96) [131]	83.64 (12.86) [135]
Hip circumference, mean (SD) [*n*]	101.35 (9.56) [82]	100.26 (9.71) [131]	101 (9.75) [135]
BMI, mean (SD) [*n*]	25.22 (4.30) [81]	25.63 (4.81) [128]	25.37 (4.54) [134]
MedD score, mean (SD) [*n*]	8.92 (1.71) [88]	9.03 (1.81) [135]	9.05 (1.81) [139]
**Smoking status, *n* (%)** - Smoker - Ex-smoker - Non-smoker	10 (11.36) 23 (26.14) 55 (62.50)	19 (13.77) 34 (24.64) 85 (61.59)	21 (14.79) 35 (24.65) 86 (60.56)
**Alcohol consumption, *n* (%)** - Nothing - ≤7 drinks/week - >7 drinks/week	44 (50) 38 (43.18) 6 (6.82)	69 (50.74) 60 (44.12) 7 (5.15)	70 (50) 62 (44.29) 8 (5.71)
Immune disease (yes), *n* (%)	5 (5.68)	7 (5.11)	7 (4.96)
Cardiovascular disease (yes), *n* (%)	16 (18.18)	22 (16.06)	24 (17.02)
Other diseases (yes), *n* (%)	6 (6.82)	12 (8.76)	13 (9.22)

BMI, body mass index; MedD, Mediterranean diet.

In terms of health conditions, 4.96% of the study population had immune-mediated diseases, while cardiovascular diseases were present in 17.02% of the study subjects. Only five participants with available anti-S IgG data reported a SARS-CoV-2-positive diagnostic test between 3 and 10 months prior to vaccination. The antibody titers were similar to those with no previous infection (**Supplementary Figure S1**).

### Antibody levels after vaccination

**Figure 1:**
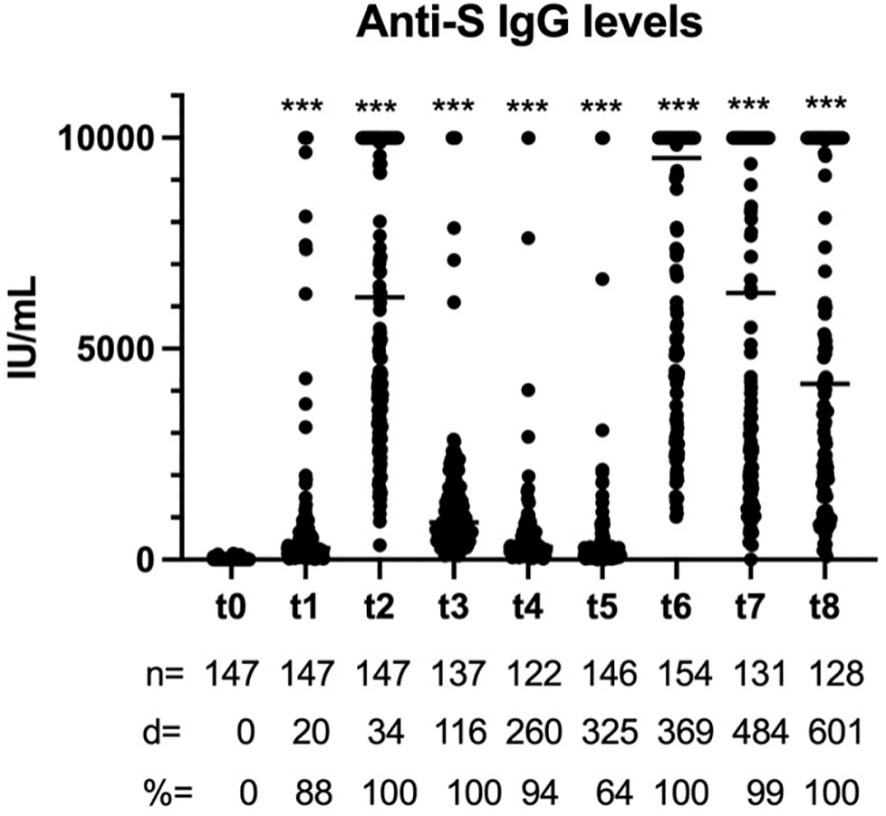
Anti-spike (S) immunoglobulin G (IgG) levels after vaccination. *n* is the sample size for each time point, *d* represents days since the administration of the first dose, and *%* denotes percentage of seroconverted individuals. ***p < 0.001.

In the primary vaccination, the highest levels of serum anti-S IgG were observed 5 weeks after the administration of the first dose and then declined progressively (**Figure 1**). Of note is that, although all individuals seroconverted after the vaccination, there was a high variability in the antibody titers observed. By the end of the initial follow-up, approximately 1 year after the primary vaccination, only 64% of individuals were still seroconverted and the antibody titers were much lower (**Figure 1**). With regard to the vaccine booster, all individuals seroconverted 1 month later, and the anti-S IgG levels were even higher than that after the primary vaccination. The titers and percentage of seroconverted individuals remained high during the follow-up (**Figure 1**).

**Figure 2:**
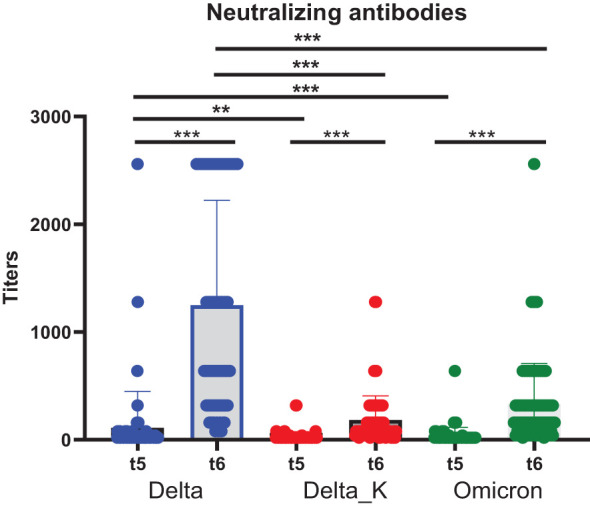
Neutralizing antibody titers after vaccination. The differences before/after the booster administration (*t*_5_ −*t*_6_) for one specific variant were assessed with the Wilcoxon matched-pairs signed-rank test. The differences between the different variant neutralizing antibody titers were analyzed with the Friedman’s test followed by Dunn’s multiple comparisons test using the Omicron variant as a reference. **p < 0.01; ***p < 0.001.

We had previously analyzed the titers of neutralizing antibodies against some of the initial SARS-CoV-2 variants after primary vaccination. In this research, we analyzed the titers of neutralizing antibodies against the Omicron, Delta, and Delta_K variants after the booster administration. The titers significantly increased after the booster administration for all variants (**Figure 2**). Moreover, the highest titers were observed against the Delta variant, both before and after the booster administration.

### Effect of exposure variables on the antibody levels after the primary vaccination

Several socio-demographic, lifestyle, and health variables were evaluated in relation to the anti-S IgG levels at the peak of the humoral response (5 weeks after vaccination). In the univariate regressions, a trend toward lower levels was observed in individuals with immune-mediated and cardiovascular diseases compared to those without disease (**Table 2**). None of these variables were retained in the stepwise multivariate models.

**Table 2:** Association between specific determinants and antibody levels at the peak of the humoral response after primary vaccination.

	Anti-S IgG			Neutralizing WT			Neutralizing Alpha			Neutralizing Beta		
	Beta	95%CI	*p*	OR	95%CI	*p*	OR	95%CI	*p*	OR	95%CI	*p*
**Age**	-0.00	-0.01 – 0.00	0.256	0.97	0.94 – 1.00	0.112	0.96	0.93 – 0.99	0.020	0.99	0.96 – 1.02	0.845
**Sex (ref. female)**	-0.21	-0.46 – 0.04	0.108	1.09	0.48 – 2.47	0.819	1.00	0.44 – 2.24	1.000	1.05	0.43 – 2.55	0.902
**Waist circumference**	-0.00	-0.01 – 0.01	0.850	1.00	0.96 – 1.03	0.951	0.98	0.94 – 1.01	0.273	0.99	0.95 – 1.03	0.728
**Hip circumference**	0.01	-0.00 – 0.02	0.263	1.01	0.97 – 1.06	0.467	1.00	0.96 – 1.05	0.784	1.01	0.96 – 1.06	0.631
**BMI**	-0.01	-0.04 – 0.02	0.700	1.00	0.89 – 1.11	0.967	0.96	0.87 – 1.06	0.465	0.98	0.87 – 1.10	0.785
**Higher Education studies (ref. no)**	-0.03	-0.38 – 0.31	0.861	1.15	0.39 – 3.38	0.791	0.51	0.16 – 1.61	0.254	1.96	0.60 – 6.41	0.264
**MedD Score**	-0.03	-0.11 – 0.04	0.435	0.97	0.74 – 1.26	0.842	1.02	0.78 – 1.33	0.863	0.88	0.67 – 1.15	0.364
**Smoking status (ref. never smoker)** - Ex-smoker - Smoker	-0.20 -0.34	-0.52 – 0.12 -0.79 – 0.09	0.216 0.126	0.50 0.44	0.17 – 1.47 0.12 – 1.59	0.209 0.217	**0.37** 0.44	**0.12 – 1.14** 0.11 – 1.65	**0.084** 0.227	0.68 **0.27**	0.22 – 2.12 **0.06 – 1.25**	0.511 **0.095**
**Alcohol consumption (ref. no consumption)** - ≤ 7 drinks/week - > 7 drinks/week	0.12 -0.29	-0.16 – 0.41 -0.86 – 0.27	0.387 0.303	1.16 0.54	0.44 – 3.02 0.11 – 2.56	0.756 0.446	1.11 0.76	0.42 – 2.92 0.16 – 3.61	0.832 0.736	1.29 2.31	0.48 – 3.49 0.52 – 10.28	0.608 0.269
**Immune disease (ref. no)**	**-0.56**	**-1.16 – 0.31**	**0.063**	2.26	0.38 – 18.65	0.324	1.82	0.29 – 11.15	0.515	0.40	0.03 – 4.78	0.471
**Cardiovascular disease (ref. no)**	**-0.31**	**-0.67 – 0.04**	**0.086**	0.77	0.25 – 2.30	0.641	0.61	0.20 – 1.82	0.381	0.40	0.11 – 1.39	0.152
**Other diseases (ref. no)**	-0.25	-0.80 – 0.30	0.372	1.00	0.22 – 4.45	0.990	1.54	0.29 – 7.99	0.607	2.29	0.48 – 10.92	0.296

Ref., reference category. Highlighted in bold if p-value<0.1. CI, confidence interval; BMI, Body Mass Index; MedD Score, Mediterranean Diet Score; WT, wild-type.

The effect of the exposure variables on the mean anti-S levels during 1 year of follow-up was also assessed (**Table 3**). Negative significant associations were found with age, smoking habit, and alcohol consumption. Moreover, positive and negative trends were observed for education level and immune-mediated diseases, respectively (**Table 3**). Considering the sex differences in alcohol metabolism, a stratified analysis was performed for the effect of alcohol on the antibody levels separately in men and women. A negative association was observed in both groups when no alcohol consumption was compared with >7 drinks/week (women: *β* = −1.28, 95%CI = −2.33 to −0.23, *p* = 0.017, *n* = 114; men: *β* = −1.87, 95%CI = −3.26 to −0.47, *p* = 0.009, *n* = 22). In the multivariate models (data not shown), the statistical significance was maintained for the smoking variable (*β* = −0.67, 95%CI = −1.34 to −0.00, *p* = 0.048) and alcohol consumption (*β* = −1.31, 95%CI = −2.15 to −0.47, *p* = 0.002). Furthermore, there was a trend toward statistical significance in the MedD score (*β* = −0.11, 95%CI = −0.24 to −0.00, *p* = 0.054).

**Table 3:** Association of specific determinants with median antibody levels at follow-up after first and third vaccine doses.

	First dose			Third dose		
	Coef	95% CI	p	Coef	95% CI	p
**Age**	**-0.01**	**-0.03 –0.00**	**0.017**	**-0.00**	**-0.01 –0.00**	**0.081**
**Sex (ref. female)**	0.09	-0.36 –0.55	0.696	0.10	-0.18 –0.38	0.488
**Waist circumference**	-0.01	-0.03 –0.00	0.231	0.00	-0.00 –0.01	0.200
**Hip circumference**	0.00	-0.02 –0.02	0.959	0.00	-0.00 –0.01	0.456
**BMI**	-0.03	-0.08 –0.01	0.131	0.00	-0.01 –0.03	0.443
**Higher Education studies (ref. no)**	**0.45**	**-0.05 –0.95**	**0.082**	0.01	-0.33 –0.36	0.948
**MedD Score**	-0.10	-0.24 –0.03	0.130	-0.01	-0.08 –0.05	0.675
**Smoking status (ref. never smoker)** - Ex-smoker - Smoker	-0.31 **-1.08**	-0.86 –0.24 **-1.70 – -0.46**	0.270 **0.001**	0.00 -0.05	-0.32 –0.34 -0.39 –0.29	0.953 0.772
**Alcohol consumption (ref. no consumption)** -≤ 7 drinks/week -> 7 drinks/week	-0.03 **-1.43**	-0.49 –0.42 **-2.20 – -0.65**	0.881 **0.000**	0.17 0.40	-0.07 –0.43 -0.08 –0.89	0.172 0.109
**Immune disease (ref. no)**	**-0.85**	**-1.86 –0.16**	**0.099**	-0.33	-1.13 –0.45	0.400
**Cardiovascular disease (ref. no)**	-0.45	-1.06 –0.16	0.150	-0.02	-0.40 –0.36	0.904
**Other diseases (ref. no)**	-0.37	-1.28 –0.52	0.412	0.05	-0.38 –0.48	0.813

Values are highlighted in bold if *p* < 0.1.

ref., reference category; BMI, body mass index; MedD Score, Mediterranean diet score; CI, confidence interval.

With regard to the neutralizing antibody titers, the following results were observed (**Table 2**): 1) for the Alpha variant, there was a negative association with age and smoking, although this did not reach statistical significance; 2) for the Beta variant, there was a borderline non-significant negative association with smoking habit; and 3) for the wild-type variant, there was no association. In the multivariate models (data not shown), the only association retained was between the neutralizing antibodies against the Alpha variant and age (*β* = −0.05, 95%CI = −0.10 to −0.00, *p* = 0.046).

### Effect of the exposure variables on the antibody levels after the booster vaccination

In the univariate regressions after the booster vaccination, the anti-S IgG levels measured at the peak of the response (*t*_6_) showed a borderline non-significant positive association with the hip and waist circumference (**Table 4**). None of these variables were retained in the stepwise multivariate models (data not shown). At the follow-up, no significant association was observed between the mean antibody levels and the different determinants measured (**Table 3**).

**Table 4:** Association between specific determinants and antibody levels after third vaccine dose.

	Anti-S IgG			Neutralizing delta			Neutralizing delta_K			Neutralizing omicron		
	Beta	95% CI	p	OR	95% CI	p	OR	95% CI	p	OR	95% CI	p
**Age**	-0.00	-0.01 –0.00	0.414	0.98	0.95 – 1.01	0.406	1.00	0.97 –1.03	0.789	1.00	0.96 –1.03	0.964
**Sex (ref. female)**	0.16	-0.09 –0.41	0.213	0.85	0.33 – 2.14	0.735	1.65	0.62 –4.39	0.314	1.40	0.53 –3.68	0.494
**Waist circumference**	**0.00**	**-0.00 –0.01**	**0.083**	1.02	0.98 – 1.06	0.173	**1.04**	**1.00 –1.08**	**0.014**	1.00	0.97 –1.04	0.718
**Hip circumference**	**0.01**	**-0.00 –0.02**	**0.053**	**1.06**	**1.01 –1.12**	**0.009**	**1.06**	**1.01 –1.11**	**0.010**	1.00	0.96 –1.04	0.861
**BMI**	0.01	-0.00 –0.03	0.173	1.10	0.98 – 1.24	0.103	1.10	0.97 –1.24	0.106	1.02	0.91 –1.13	0.712
**Higher Education studies** **(ref. no)**	0.02	-0.23 –0.29	0.839	1.55	0.54 – 4.46	0.409	0.94	0.32 –2.69	0.909	1.00	0.34 –2.90	0.994
**MedD Score**	-0.03	-0.09 –0.02	0.248	0.84	0.66 – 1.07	0.177	0.94	0.74 –1.18	0.613	**0.74**	**0.58 –0.96**	**0.023**
**Smoking status (ref. never smoker)** - Ex-smoker - Smoker	0.19 0.11	-0.06 –0.45 -0.20 –0.44	0.140 0.476	0.78 1.13	0.31 – 1.94 0.32 – 3.96	0.599 0.837	1.39 0.84	0.55 –3.48 0.27 –2.59	0.481 0.770	1.14 1.37	0.44 –2.91 0.44 –4.22	0.784 0.579
**Alcohol consumption (ref. no consumption)** - ≤ 7 drinks/week - > 7 drinks/week	0.02 0.17	-0.19 –0.25 -0.37 –0.72	0.801 0.525	0.86 0.26	0.33 – 2.23 0.02 – 2.57	0.767 0.251	1.74 0.95	0.67 –4.52 0.14 –6.40	0.252 0.965	1.28 5.5	0.49 –3.34 0.56 –53.98	0.602 0.143
**Immune disease (ref. no)**	0.06	-0.43 –0.55	0.807	2.48	0.38 – 15.92	0.338	1.00	0.20 –4.81	1.000	1.75	0.39 –7.79	0.459
**Cardiovascular disease (ref. no)**	-0.01	-0.31 –0.28	0.938	0.95	0.33 – 2.73	0.937	1.18	0.40 –3.46	0.759	1.11	0.39 –3.14	0.841
**Other diseases (ref. no)**	0.29	-0.08 –0.67	0.131	0.63	0.15 – 2.52	0.515	1.11	0.25 –4.85	0.888	0.79	0.18 –3.44	0.754

Values are highlighted in bold if *p* < 0.1.

ref., reference category; CI, confidence interval; BMI, body mass index; MedD Score, Mediterranean diet score.

With respect to the neutralizing antibodies for the predominant variants in that phase of the pandemic, the following associations were observed (**Table 4**): 1) for the Delta variant, there was a positive association with hip circumference; 2) for the Delta_K variant, there were positive significant associations with the hip and waist circumference; and 3) for the Omicron variant, there was a significant negative association with the MedD score. In the multivariate models, the following associations were retained (not shown): anti-Delta antibodies with hip circumference (OR = 1.07, 95%CI = 1.01–1.12, *p* = 0.008); anti-Delta_K antibodies with hip circumference (OR = 1.06, 95%CI = 1.01–1.11, *p* = 0.007); and anti-Omicron with the MedD score (OR = 0.74, 95%CI = 0.58–0.96, *p* = 0.023).

## Discussion

In the present study, factors that influence the BNT162b2 vaccine response of both the initial vaccination and the booster dose were explored.

In our cohort, the vaccine seroconversion curve was highest at 5 weeks after the first dose; however, the titers and the percentage of seroconverted individuals declined over time, prior to the administration of the booster dose, and a high variability on the antibody titers was observed. This is consistent with previous results in other cohorts (11–14). In contrast to these studies, some of them performed in health workers, higher antibody titers were not observed in individuals with a previous history of COVID-19 infection, nor even before the vaccine administration. This could be due to the low number of pre-exposed subjects in our cohort and the long period of time between the infection and the measurement of antibodies. However, other studies did not show differences in the vaccine-induced antibody levels between unexposed and previously SARS-CoV-2-exposed health workers (15).

After the booster administration, the antibody titers increased and remained high during the 9 months of follow-up, in consonance with other studies (11, 14, 16). These results suggest that a third dose is necessary to maintain the high titers of antibodies and, presumably, the protection in the population. The possible impact of the third dose on disease transmission has yet to be investigated. However, currently, in Spain and many other countries, a third dose is only recommended for risk groups.

Although the booster dose administered had the sequence of the wild-type variant (17–19), the neutralizing antibody levels increased significantly after this dose, even against the more recent and different variants, e.g., Omicron and Kappa, as previously reported (20). However, the titers were significantly higher for the earlier variant, Delta.

In our cohort, the mean antibody levels at follow-up had a negative association with age after the initial dose, and a similar trend was observed after the booster dose. Moreover, age was negatively associated with the neutralizing antibody titers against the Alpha variant. This result is in consonance with other cohorts (8, 12, 21–23). However, we did not find any association with age at the peak of the antibody response, i.e., 5 weeks after the first vaccination. This may be due to the fact that our study subjects were health workers, with an age range of 24–66 years, meaning the extreme age groups were excluded from our cohort. Of note is that, in most studies, the strongest age-related association with antibody levels has been observed in the age group >65 years (8, 12). Furthermore, we did not find any association of the antibody levels with sex. This is in consonance with previous studies (11, 14, 22), but in contrast to others (8, 12, 21, 23) that found men to have lower antibody titers than women. Thus, more studies are necessary to confirm this finding.

Lifestyle habits, smoking status, and, to a lesser extent, alcohol consumption were negatively associated with the antibody response to the BNT162b2 vaccine in our cohort, as previously reported for COVID-19 (21, 22) and other vaccines [reviewed in (24)]. Smoking reduced the percentages of cytotoxic CD8^+^ T cells, total lymphocytes with granzyme B and perforin, and lymphocytes with KLRB1 (CD161) expression (25). Similarly, some studies suggest that alcohol consumption has deleterious effects on the response to infection, with potential mechanisms that include alterations in the migration of leukocytes to sites of infection, functional abnormalities in T and B cells and other leukocytes, and changes in cytokine expression [reviewed in (26)]. Notably, it has been reported that women are more susceptible to alcohol-related effects on the inflammatory and immune responses [reviewed in (27)]. However, a negative association was observed in both sexes, which was actually slightly stronger in men.

Several studies have directly examined gender differences in the effects of alcohol on inflammatory and immune responses, reporting that women exhibit greater sensitivity to alcohol than men.

We found that those with previous immune-mediated diseases had borderline non-significant lower levels of anti-S IgG levels at the peak of the seroconversion curve, as reported previously in individuals taking immunosuppressants (8, 21, 28) or in those with immunosuppression (12). The deleterious effect of comorbidities related to the immune system on the vaccine response appears quite logical. However, we did not observe such an effect during follow-up or after the booster dose, nor with regard to the neutralizing antibody titers, suggesting that, overall, the vaccine response may be sufficient or not different from the response in individuals without those diseases, at least after a booster dose has been administered. This is in consonance with previous studies (29, 30).

No significant association was found between the humoral response to the vaccine and BMI; however, after the booster dose, a consistent positive association was observed between the hip or waist circumference and the levels of anti-S IgG or neutralizing antibodies. In agreement with this, a negative association was observed between the titers of the anti-Omicron neutralizing antibodies and the MedD score, a healthy nutritional pattern largely seen as a protective factor for obesity (31). Notably, a recent cross-sectional study has shown that the direction of the association of body fat with the COVID-19 vaccine-induced antibody response is dependent on sex, being negative in men and positive in women (32). Around 80% of our cohort were female health workers, which could probably explain our result. In this regard, in another cohort of women receiving a different anti-SARS-CoV-2 vaccine, a positive association of the antibody titers with obesity has been shown (33).

Among the limitations of this study are the relatively small sample size and the choice of health workers as the subjects, who may not be representative of the general population as they have generally better health and lifestyle, resulting in a more robust immune response. In addition, only antibodies were measured; however, it is well accepted that the cellular response plays an important role in vaccine-induced immunity. In addition, clinical and lifestyle information was collected by means of retrospective auto-administered questionnaires, with a potential information bias. This study also has important strengths. Our data provided information on antibody dynamics in a controlled population, with similar blood extraction timings and a reasonable amount of follow-up. In addition, both anti-S and neutralizing antibodies were measured against different variants, providing a good picture of the immunogenicity of BNT162b2.

## Conclusions

In health workers vaccinated with BNT162b2, the antibody titers and the percentage of seroconverted individuals declined 1 year after the primary vaccination; however, they remained high after the booster administration. Age, immune-mediated comorbidities, smoking habit, and alcohol consumption were negatively associated with the antibody response. In contrast, waist and hip circumferences were positively associated with the antibody response after vaccination.

## Data Availability

The raw data supporting the conclusions of this article will be made available by the authors, without undue reservation.
