# Supplementary material for: Negative associations of age and lifestyle factors with the antibody response to the COVID-19 vaccine BNT162b2 in health workers from Spain
Source: Front Immunol. 2025 May 30;16:1590939. doi: 10.3389/fimmu.2025.1590939 (PMC12163240; doi:10.3389/fimmu.2025.1590939)
Supplement: Supplementary file 1 [file Table1.docx]

Supplementary Figure 1. Baseline antibody levels according to previous infection status.

The boxes represent the median, and 25th and 75th percentiles (lower and upper hinge respectively) of anti-S IgG levels in individuals without (NO, n=90) or with (YES, n=5) a history of COVID-19 infection before vaccination. Differences (p-value) were assessed with the Mann-Whitney test as values did not follow a parametric distribution.
